# Cell Death by Holocrine Secretion: The Final Step of Epithelial Differentiation in Sebaceous Glands

**DOI:** 10.3390/cells15121058

**Published:** 2026-06-10

**Authors:** Leopold Eckhart, Supawadee Sukseree, Heinz Fischer

**Affiliations:** 1 Department of Dermatology, Medical University of Vienna, 1090 Vienna, Austria; 2 Division of Cell and Developmental Biology, Center for Anatomy and Cell Biology, Medical University of Vienna, 1090 Vienna, Austria; heinz.fischer@meduniwien.ac.at

**Keywords:** skin, sebaceous gland, programmed cell death, holocrine secretion, exocrine gland, deoxyribonuclease, epithelial cell, keratinocyte, lysosome, autophagy

## Abstract

Sebaceous glands consist of epithelial cells, known as sebocytes, that undergo differentiation to deliver the components of sebum into the sebaceous duct and eventually to the hair and skin surface. The final step of the terminal differentiation program is called holocrine secretion because the entire cell content is converted into sebum. Holocrine secretion is a mode of programmed cell death, which involves the degradation of the nucleus and other organelles and the rupture of the cell membrane. Here, we review the current knowledge of differentiation-associated death of sebocytes and discuss open questions regarding its mechanism and functions. In vivo studies have provided evidence for degradation of nuclear and mitochondrial DNA by lysosomal deoxribonuclease 2 (DNase 2), indicating a key role of lysosomes in holocrine secretion. We discuss the influence of tight junctions on the spatial localization of holocrine secretion within glands, the regulation of holocrine cell death by autophagy and potential mediators of membrane lysis. Further studies of holocrine secretion are needed to fully uncover its molecular control and to determine potential clinical implications.

## 1. Introduction: Cell Death in the Skin

The skin consists of an epithelial compartment, the dermis and the hypodermis. Epithelial cells form the epidermis, hair, nails and glands, such as eccrine and apocrine sweat glands, and sebaceous glands. The latter are mostly associated with hair follicles, forming the so-called pilosebaceous unit, but there are also sebaceous glands independent from hairs, such as Meibomian glands at the eyelid [1]. The function of sebaceous glands is the production of sebum, an oily secretion important for the maintenance and defense of normal hair and skin surfaces [2,3,4,5,6]. In addition, specialized sebaceous glands contribute to the stabilization of the tear film (depending on Meibomian glands) and to pheromone-based communication (depending on preputial glands). The pathophysiological relevance of sebaceous glands for benign and malignant tumors [7,8,9,10], acne [11,12,13,14], inflammatory skin diseases [15,16] and skin care [5,17] makes them important subjects of biomedical research projects.

The epidermis and the skin appendages are not static tissue structures but undergo continuous or periodic (in the case of hair) renewal. This epithelial turnover and damaging effects from the environment lead to significant rates of cell death in the skin [18]. Cell death is induced by exogenous and endogenous stimuli in the skin. Ultraviolet radiation and other types of physical stress, noxious chemicals and infections by bacteria and viruses can cause cell death. Both programmed cell death in the form of apoptosis, pyroptosis and necroptosis, and uncontrolled cell death in the form of necrosis occur in the skin. Endogenous triggers of cell death are important for embryonic development and cell differentiation. The latter term refers to the step-wise conversion of a proliferating, functionally less specialized cell into a mature cell adapted to a specific function. The barrier function of the epidermis depends partly on the extensive cross-linking of intracellular proteins, which is incompatible with cell survival [19,20]. Likewise, the growth of hair and nails requires the cornification of their constituent keratinocytes, also referred to as trichocytes and onychocytes, respectively, resulting in dead but stable and functional structures [21]. In the sebaceous glands, epithelial cells must die to form sebum through holocrine secretion [22], as will be discussed in detail below. To avoid the impression that all types of epithelial differentiation lead to cell death, we point to differentiated sweat gland secretory cells, which are fully functional when they are alive, and exhibit slow turnover [23]. However, some skin epithelial cells undergo programmed cell death to fulfill their function [24]. While cell death by cornification, also called “corneoptosis”, has been covered in previous reviews [19,20], holocrine secretion has not been comprehensively discussed as a mode of cell death, although the importance of cell death for the execution of holocrine secretion was highlighted by recent papers [24,25]. The aim of the present paper is to summarize what is known about the process of holocrine cell death in sebaceous glands and to discuss existing gaps of knowledge pertaining to its mechanism, regulation and function.

Holocrine secretion was not discussed in the recommendations published by the Nomenclature Committee on Cell Death (NCCD) [26]. The NCCD suggested “to keep PCD (programmed cell death) and terminal differentiation conceptually well discriminated from each other. Indeed, dead cells are disposed of (and hence cease to have a function) in the course of PCD. Conversely, when terminal differentiation involves cellular demise, as in the case of cornification, dead cells become integral part of a tissue (and hence mediate a specific physiological function)” [26]. Here, we will use the term “cell death” to simply refer to the “end of life of a cell” without a “conceptual” exception for terminal differentiation. Holocrine secretion involves cellular demise, and cells do not “become integral part of a tissue.”

## 2. Sebaceous Glands: Structure and Functions

Sebaceous glands are built by epithelial cells known as sebocytes [27,28,29]. However, Langerhans cells and non-melanogenic melanocytes are present within sebaceous glands [30,31]. Although the biological roles of sebocytes are mainly determined by their autonomous differentiation, interactions with other cell types in their environment are also important. For example, sebocytes were shown to promote the differentiation of T helper 17 cells [32] and to communicate with innate lymphoid cells [33] and nerves [34]. Recently, the outer sebaceous duct was reported to have the highest prevalence of CD8+ resident memory T cells across all skin niches, possibly contributing to the inflammatory memory of atopic dermatitis and psoriasis [35].

**Figure 1:**
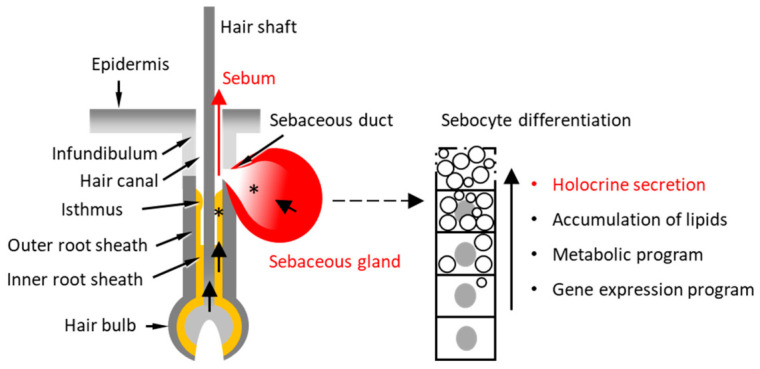
Structure of a sebaceous gland associated with a hair follicle, forming the pilosebaceous unit. Important elements are labeled. Sites of terminal differentiation of epithelial cells are indicated by thick arrows and the positions of holocrine secretion and disintegration of the inner rooth sheath are indicated by asterisks. Sebocyte differentiation is depicted on the right, with cells represented by squares, nuclei by grey ovals, and lipid droplets by circles. This figure is adapted from a paper recently published by us under a Creative Commons CC-BY 4.0 license (open access) license [41].

Sebaceous glands consist of acini in which sebocytes proliferate within the basal layer and differentiate upon detachment from the basement membrane (Figure 1). Terminal differentiation of sebocytes culminates in programmed cell death, known as holocrine secretion, to produce sebum. The latter leaves the gland through the sebaceous duct, which is formed by epithelial cells undergoing a distinct differentiation program [36,37,38]. Most sebaceous glands are associated with hair follicles, and sebum enters the hair canal, from where it is distributed onto the hair and skin surface. Special types of these glands, such as preputial glands and Meibomian glands, are not physically linked to hair. In line with the specialized functions, the secretions of preputial glands and Meibomian glands have unique molecular compositions [39,40]. Nevertheless, according to current knowledge, the basic regulation of cell differentiation and holocrine secretion is conserved in specialized sebaceous glands, so that they can be used as models for the study of basic principles of sebocyte differentiation in vivo.

Sebum composition differs among species of mammals [6,42]. To some extent, these differences represent adaptations of sebaceous glands to different environments and intra-species communication. Sebaceous glands have degenerated in several phylogenetic lineages of mammals including cetaceans, the hippopotamus, rhinos, elephants, sirenians and the naked mole rat [42,43,44].

The functions of sebum include the protection of the hair coat against water, the improvement of the barrier function of the skin, and the defense against microbes. In the case of the meibomian gland, the sebum (also called meibum) helps to maintain the tear film of the eye. The physical barrier-related functions of the sebum depend almost entirely on the high lipid content. The synthesis and interactions of sebum lipids have been reviewed extensively [17,22,45,46,47]. Lauric acid, which is one of several free fatty acids of human sebum, exhibits antibacterial activity against Gram-positive bacteria such as *Cutibacterium acnes*, previously known as *Propionibacterium acnes* [48]. Free fatty acids induce the production of antimicrobial proteins such as beta-defensins [49] and cathelicidin [50], but they inhibit the antimicrobial protein RNase 7 [51]. Another type of antibacterial components of sebum are histones such as histone H4 and possibly histone-derived peptides [52,53]. Interestingly, histones are able to bind to lipid droplets, from which they are released upon exposure to bacterial lipopolysaccharide or lipoteichoic acid [54]. However, it has not been determined yet whether histones interact with lipid droplets in sebocytes or in sebum. Taken together, the functions of sebum are determined not only by lipids but also by other cell components released by holocrine cell death.

Research on sebaceous glands depends on several complementary approaches. Histological and ultrastructural studies of sebaceous glands in human clinical samples and samples from various other species have revealed essential basic insights. Targeted gene deletion methods have enabled mechanistic studies of gene functions in sebaceous glands of the mouse [55,56,57]. Appropriate protocols for the study of mouse sebaceous glands have been published [58]. In vitro, sebocyte cell lines are important research tools [59]. Most recently, three-dimensional models of sebaceous glands [60,61,62,63] have allowed reseachers to replicate critical sebocyte differentiation steps in vitro.

## 3. Differentiation of Sebocytes

The transcriptional regulation of early development and differentiation events of sebaceous glands has been extensively investigated [9,64,65,66,67,68,69]. Recent studies have also provided an overview of the later differentiation steps in sebaceous glands including the production of lipid droplets, which is arguably the main feature of sebocyte differentiation [14,70,71,72,73,74,75]. Lipid-binding proteins and enzymes of the lipid metabolism, such as acyl-CoA wax alcohol acyltransferase 1 (AWAT1), elongation of very long-chain fatty acids 3 (ELOVL3), ELOVL4, fatty acid desaturase 1 (FADS1), FADS2, and perilipin-2 (PLIN2) are useful differentiation markers of sebocytes. Notably, the cells of the sebaceous duct undergo a specific differentiation program that does not lead to holocrine secretion [38,40].

Keratins are major markers of skin epithelial cells including those of the sebaceous glands [71]. Different from epidermal keratinocytes, sebocytes express Krt7 and Krt79. The latter is one of the keratins characterized most recently. Keratin Krt79 is specifically expressed in the infundibulum of the hair follicle, the sebaceous duct and the sebaceous gland [57,76]. When sebaceous gland cells of the basal layer start differentiation, the expression is shifted from Krt5 to Krt79 [57]. Krt79 heterodimerizes with Krt14 in sebocytes, and deletion of Krt79 leads to destabilization of Krt14. Krt79-deficient mice lost meibomian glands in the eyelid, and the loss of lipid-dependent lubrication affected the corneal surface, whereas the growth and cycling of hair was not defective [57].

The keratin intermediate filament networks of neighboring sebocytes are connected by desmosomes. In addition, gap junctions and tight junctions (TJs) are present between sebocytes [77,78]. Desmosomes increase in number during sebocyte differentiation [79], and functional TJs locate to a single layer of differentiated sebocytes (see Section 4 for details). Together, intracellular and intercellular proteins contribute to control of sebaceous gland structure and cell turnover.

## 4. Holocrine Secretion: Mechanism and Regulation

### 4.1. Spatial Control of Holocrine Secretion

**Figure 2:**
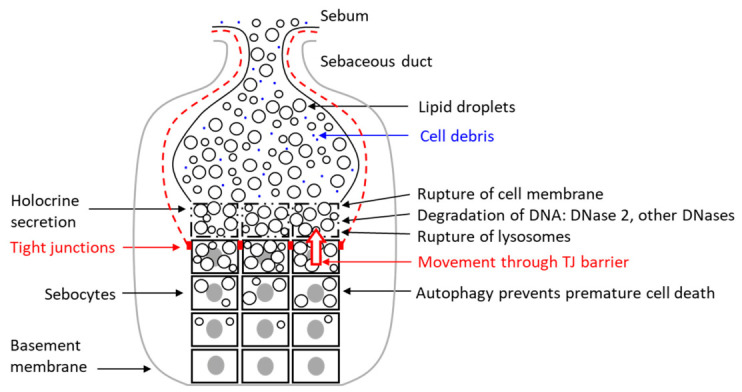
Holocrine secretion occurs at the end of sebocyte differentiation within sebaceous glands. Cells are indicated with black lines representing the cell membranes for a part of the gland. Nuclei are depicted as grey ovals. Lipid droplets are indicated by circles. Other cell components released from dying cells are depicted as blue dots. The positions of the basement membrane and tight junctions are indicated by grey continuous and red discontinuous lines, respectively. TJ, tight junction.

As the last step of terminal differentiation of sebocytes, the spatial localization of holocrine secretion is tightly controlled. It depends on preceding intracellular remodeling during differentiation and on conditions outside of the cell or at the surface of the cell. Although the initiation of holocrine secretion is not fully understood, recent evidence shows that it occurs outside the TJ barrier of sebaceous glands [80] (Figure 2). In this study, TJ formation was demonstrated in a three-dimensional sebaceous gland model consisting of immortalized human sebocytes and in murine sebaceous glands in vivo [80]. The deletion of the TJ protein claudin-1 severely disturbed holocrine secretion in a mouse model [80]. The TJ barrier of induced skin-specific claudin 1 (Cldn1) conditional knockout mice was leaky in a lanthanum permeation assay assessed under the electron microscope, and the degradation of the plasma membrane and the nucleus of terminally differentiated sebocytes was incomplete, leading to the aberrant accumulation of sebocyte remnants in sebaceous ducts. Notably, the diffusion of proteins was not possible in the absence of claudin-1, suggesting that remaining junctions positive for ZO-1 maintain part of the functions of TJs, which is comparable to the phenotype of claudin-1 knockout in the interfollicular epidermis [81]. Recently, the expression of claudin-1 was reported to be decreased in acne vulgaris [82]. The potential impact of this decrease on the regulation of holocrine secretion remains to be investigated.

### 4.2. Intracellular Initiation of Holocrine Secretion: Roles of Lysosomes

The initiation of sebocyte programmed cell death is only partly understood at present. Apoptotic mechanisms and lysosome-dependent processes have been discussed as alternative initiators of cell death in sebaceous glands. Soon after the characterization of terminal deoxynucleotidyl transferase dUTP nick end labeling (TUNEL)-positive DNA fragmentation as a basic feature of apoptosis, the detection of DNA fragments was regarded a sign of apoptotic or apoptosis-like cell death in the differentiated compartments of the sebaceous gland, the hair follicle and the epidermis [83,84,85]. Moreover, the differentiation of sebocytes leads to features of apoptosis in vitro [86]. However, nuclear DNA breakdown is a feature shared by many modes of cell death, and further research showed that sebocyte terminal differentiation is mechanistically different from apoptosis in vivo. Interestingly, the so-called “cell death inducing DNA fragmentation factor A (DFFA)-like effector A” (CIDEA) is highly expressed in sebocytes. CIDEA is anchored in the phospholipid layer of lipid droplets [87] and controls lipid storage and secretion in sebaceous glands [88], but this role is not related to apoptosis. The prototypic executioner of apoptotic cell death, caspase-3, is not involved in differentiation-associated death of sebocytes but even plays a role in sebocyte proliferation by cleaving α-catenin to activate YAP-dependent signaling [89].

Similar to holocrine secretion, cornification of keratinocytes was previously discussed as a subtype of apoptosis. However, caspase-14, a well-established marker of epidermal keratinocyte differentiation, does not function as an apoptotic protease [18]. In early studies, caspase-14 was detected also in sebaceous glands [90,91], but later scRNA-seq studies, which are not affected by potential cross-reactivity of probes, indicate that the expression level of caspase-14 is minimal in sebocytes [72,92], supporting the clear distinction between sebocyte and interfollicular keratinocyte differentiation. Proteases other than caspase-14 and protease inhibitors contribute to the control of terminal differentiation of sebocytes and can be detected in the sebum [93,94,95]. However, their specific roles in sebocyte differentiation remain to be determined.

**Figure 3:**
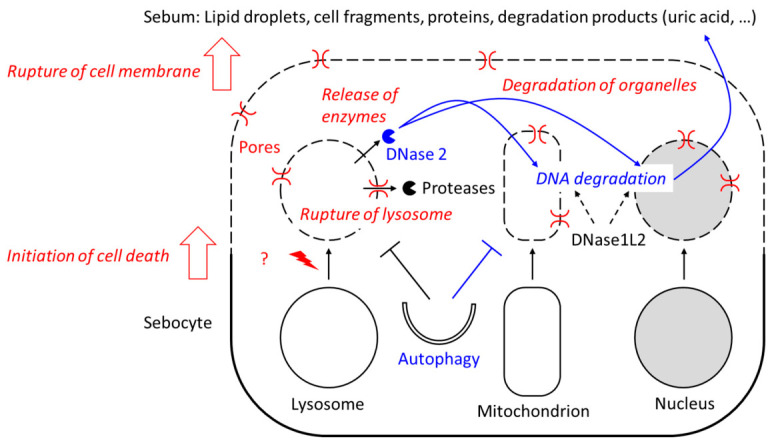
Intracellular events leading to sebocyte death in the course of holocrine secretion. The schematic diagram summarizes both processes that have been validated by targeted gene deletion studies in vivo (blue lines and fonts) and processes that are discussed as hypotheses in the main text (red fonts). Autophagy was shown to suppress premature DNA degradation in sebocytes, and the removal of damaged lysosomes was proposed but not confirmed as the underlying mechanism. A question mark indicates that the mechanism of initiating cell death is not known. The sequence of events leading from intact organelles to fragments and degradation products is depicted from the bottom to the top. Lipid droplets and several other subcellular structures are not shown for simplicity.

Lysosome-dependent cell death is initiated by lysosomal membrane permeabilization (LMP), which leads to the release of lysosomal proteases and other enzymes into the cytoplasm [26]. Lysosomes contribute to skin epithelial cell differentiation [96,97], and some evidence argues for a specific role in sebocytes [98]. Ultrastructural studies have shown that lysosomes are abundant in sebocytes [99,100,101,102]. The lysosomal membrane protein LAMP1 is readily detectable in basal and intermediate differentiated sebocytes, but it disappears in terminally differentiated sebocytes [56]. Targeted gene deletion studies revealed that the lysosomal enzyme DNase 2 degrades nuclear and mitochondrial DNA in sebaceous glands [56], indicating that DNase 2 is released from lysosomes (Figure 3). A role of uptake of DNA into lysosomes and intra-lysosomal activity of DNase 2 in the course of nucleophagy [103] or mitophagy can be excluded because LAMP1-positive lysosomes disappear before nuclear DNA is degraded, and suppression of autophagy by deletion of Atg7 does not abrogate DNA breakdown [56]. Thus, alterations of lysosomes—probably in the form of membrane disintegration—occur in the course of holocrine secretion, and at least one lysosomal enzyme (DNase 2) contributes actively to holocrine secretion-associated cell death.

### 4.3. Breakdown of Mitochondria and Other Cytoplasmic Organelles

In addition to lysosomes (see above), mitochondria, the endoplasmic reticulum, the Golgi apparatus, and peroxisomes are present in viable sebocytes [104]. Ultrastructural studies have shown degenerating organelles in sebaceous glands [102,105]. Autophagy is active in sebaceous glands (see Section 4.6) and likely contributes to the degradation of organelles [41,102,105]. The comparative investigation of wildtype mice and mice lacking DNase 2 in epithelial cells has revealed that mitochondrial DNA is at least partially degraded by DNase 2 [56]. It has not been determined yet whether this degradation occurs in lysosomes upon autophagy of mitochondria (mitophagy) or in the cytoplasm upon disruption of lysosomal and mitochondrial membranes. In the latter scenario, which appears more likely considering the points discussed in Section 4.4 and Section 4.6, the cytoplasmic pH needs to drop sufficiently to allow catalytic activity of DNase 2 [56]. The pore-forming protein gasdermin A (GSDMA), which is abundant in sebaceous glands [106], preferentially targets mitochondrial membranes [107], suggesting that GSDMA is candidate effector of mitochondrial breakdown during holocrine secretion.

Interestingly, targeted elimination of mitochondria affects sebaceous glands in several ways, which are only partly linked to terminal sebocyte differentiation, namely alterations in the expression of stem cell markers, senescence markers and lipogenesis genes, the aberrant accumulation of lipids, inflammation and defects in the delivery of sebum to the skin surface [108]. Further studies involving the blockade of mitochondrial proteins or putative regulators of mitochondrial degradation are needed to clarify the roles and dynamics of mitochondria in sebaceous glands.

### 4.4. Breakdown of the Nucleus and Degradation of DNA During Holocrine Secretion

The mechanism of DNA degradation in sebocytes was systematically investigated by comparing the fate of endogenous DNA in wildtype mice and mice lacking DNase 1, DNase1-like 2 or DNase 2 [56]. DNA degradation was impaired upon the deletion of *Dnase2a* specifically in skin epithelial cells including sebocytes. As DNase 2 is located to lysosomes, it is remarkable that the lysosomal membrane protein LAMP1 disappears in sebocytes that undergo DNA degradation. This coincidence is compatible with a loss of lysosomal integrity and the release of DNase 2 [56]. The suppression of autophagy in epithelial cells leads to the premature degradation of the nucleus during sebocyte differentiation. The co-deletion of both DNase 2 and the autophagy regulator Atg7 resulted in retention of nuclear DNA, similar to the result of DNase 2 deletion alone [56]. Thus, autophagy is not required for nuclear DNA degradation whereas DNase 2 is essential for complete DNA degradation during holocrine secretion.

When DNA degradation was impaired by the deletion of DNase 2, DNA was increased in the sebum. By contrast, uric acid, which is produced from free nucleotides, was decreased, suggesting that the nucleotide pool of differentiated sebocytes is supplied by DNA breakdown and supports the formation of uric acid [56]. DNase 2 is secreted as part of the sebum and remains active on the skin surface. Indeed, DNase 2 is the main DNA-degrading enzyme on the skin surface, with sebum and cornifying epidermal keratinocytes being the sources of this enzyme [109].

DNase 2 cooperates with other DNases during holocrine secretion. DNase1L2 is a likely contributor to sebocyte DNA degradation, because it is highly abundant in sebaceous glands [72]. In epithelial cells of the epidermis, cooperation of DNase1L2 and DNase 2 has been experimentally confirmed [110]. Like other members of the DNase 1 family, DNase1L2 generates DNA fragments with free 3′-OH ends, and such DNA ends are detectable by the TUNEL reaction in sebocytes undergoing holocrine secretion. However, TUNEL-positivity is not abrogated by the deletion of DNase1L2 in mice, suggesting that an additional enzyme DNA-degrading enzyme is active in sebaceous glands [56].

### 4.5. Rupture of Membranes During Holocrine Secretion

One of the characteristic features of holocrine secretion is the rupture of the cell membrane. Accordingly, the central portion of the sebaceous gland is often referred to as the “necrotic zone”. Research of the past twenty years has shown that, besides the exogenous destruction of a cell during classical necrosis, several pathways of intrinsically controlled necrosis exist, known as necroptosis and pyroptosis [26,111]. The molecular mechanisms of pore formation in these cell death modes are also candidate mechanisms for the rupture of the cell membrane during holocrine secretion. Here, we provide a brief overview on candidate pore-forming proteins, but it is important to note that the mechanism of membrane rupture during holocrine secretion has not been established yet, and an uncharacterized mechanism may operate independently of the candidate proteins in sebaceous glands.

During necroptosis and pyroptosis, mixed lineage kinase domain-like pseudokinase (MLKL) and gasdermins, respectively, form holes in the cell membrane [112]. The formation of these pores is followed by extensive rupturing of the membrane by ninjurin (NINJ1) [113,114,115,116]. Gasdermins are also able to permeabilize the mitochondrial membrane [106,117]. The autophagy-related protein ATG9A appears to counteract some of the pore-forming processes in the plasma membrane [118].

Preliminary evidence suggests that members of the aforementioned families of gasdermin and ninjurin proteins are expressed in sebocytes, whereas the expression levels of MLKL are low [72,74]. GSDMA is predominantly expressed in epithelial cells of the skin and its expression is upregulated during cell differentiation [106]. GSDMA was demonstrated to be cleaved by bacterial proteases leading to pyroptosis in the course of anti-bacterial defense [119,120]. Nevertheless, an additional role of GSDMA in epithelial cell differentiation is conceivable. Mutations of Gsdma3, one of three GSDMA paralogs in the mouse, cause defects of sebaceous glands and hair follicles [121,122,123]. However, knockout of all three GSDMA paralogs did not lead to an obvious skin phenotype in mice [124], suggesting that the phenotype of Gsdma3 mutants [121] may be due to gain of function. A detailed investigation of sebaceous glands of GSDMA-deficient mice has not been published yet.

Besides GSDMA, also ninjurin (NINJ1) is expressed in differentiated sebocytes [74]. The protein levels of NINJ1 are higher in the skin than in all other organs except the ileum, and by immunohistochemistry it was detected in sebocytes [125]. Upregulation of NINJ1 in response to X-irradiation of keratinocytes was reported [126], but the function of NINJ1 in skin epithelial cells and particularly sebocytes has not been tested yet.

### 4.6. Roles of Autophagy in Sebocyte Differentiation and the Control of Holocrine Secretion

Autophagy is an intracellular degradation mechanism that is active during the differentiation of skin epithelial cells [41,102,103]. Although autophagy contributes to some forms of cell death [126,127], it is not considered a mechanism of cell death on its own [26]. In sebaceous glands, holocrine secretion is preceded by active autophagy which affects the composition of the sebum [102,128,129,130]. Suppression of autophagy results in a sebaceous gland phenotype that can be recognized by regular histology. Autophagy-related gene knockouts in mouse models have shown that autophagy-deficient sebocytes undergo premature loss of the nucleus and the entire differentiated compartment of the sebaceous glands becomes more eosinophilic in hematoxylin-eosin staining [56,128]. Both phenomena are blocked by the co-deletion of the lysosomal enzyme DNase 2, suggesting that autophagy extends the survival of sebocytes during differentiation, while DNase 2-mediated DNA degradation influences, by an unknown mechanism, the affinities of sebaceous gland structures to eosin and hematoxylin. We have proposed that autophagy eliminates damaged lysosomes through lysophagy [131,132] in normal sebaceous glands, whereas lack of autophagy-dependent quality controls allows defective lysosomes to trigger premature cell death [56].

An alternative mechanism by which autophagy-related proteins such as ATG5 can contribute to the quality control of lysosomes depends on “conjugation of ATG8s to single membranes” (CASM) [133]. This process is triggered by the release of calcium ions and causes the recruitment of endosomal sorting complex required for transport (ESCRT), leading to the repair of damaged lysosomal membranes [134]. The potential roles of CASM in the sebaceous glands remain to be investigated.

Recently, autophagy-related 9A (ATG9A) was shown to support lysosomal repair in cooperation with ADP ribosylation factor interacting protein 2 (ARFIP2) through lipid transfer [135]. ATG9A also facilitates the closure of autophagosomes, enables lipid mobilization from lipid droplets and protects the plasma membrane from permeabilization [117,136,137]. Interestingly, an incompletely characterized paralog of ATG9, ATG9B is predominantly expressed in the skin and skin appendages including the sebaceous gland [74], suggesting that its role in sebaceous glands is a worthwhile topic of future research.

## 5. Differentiation-Associated Cell Death in the Inner Root Sheath: Impact on Sebum Composition

In sebaceous glands associated with hair follicles, sebum is excreted through the sebaceous duct into the hair canal. At this point, it mixes with the debris of epithelial cells undergoing programmed cell death in the inner root sheath (IRS) and the companion layer of the hair follicle. The IRS consists of three concentric layers, namely IRS cuticle, Huxley’s layer and Henle’s layer. The companion layer surrounds the Henle’s layer and is considered part of the outer root sheath (ORS) in most publications, whereas some papers assign it to the IRS. The IRS supports the morphogenesis of the hair shaft and its cellular components must disintegrate to allow the hair shaft to emerge from the skin surface [138,139]. The differentiation-associated death of IRS and companion layer cells occurs at different levels of the hair follicle, and the resulting debris fuses with the holocrine secretion products of sebocytes [140].

The modes of cell death in the IRS and companion layer are only partially characterized. The deletion of DNase1L2 led to the aberrant retention of nuclear DNA in the IRS cuticle [56], whereas nuclear DNA degradation in the isthmus of the follicle was blocked by the deletion of DNase 2 [56]. According to histological studies, the epithelial cells of the isthmus may be derived from the ORS and possibly represent terminally differentiated cells of the companion layer [140,141,142]. The programmed death of IRS and companion layer cells shares several features with holocrine secretion in the sebaceous glands. For mechanistic comparisons, focused studies on this often neglected part of the pilosebaceous unit are needed.

## 6. Holocrine Cell Death in Normal and Diseased Skin: Potential Dermatological Implications

Cell death is an essential step of the secretion of sebum from normal sebaceous glands. The potential dysregulation in diseases has not been explored in detail yet. Here, we discuss changes of cell death in sebaceous gland abnormalities and effects of holocrine cell death on sebum properties important for the etiology of skin diseases.

Acne is associated with defects in epithelial cell differentiation in the upper pilosebaceous unit [11,12,143,144]. The aberrant accumulation of cornified cells in the infundibulum leads to the formation of comedones, which contain DNA diffusely labeled on histological sections [14]. The dysfunction of degradative processes during holocrine secretion may contribute to the initiation and the progression of the disease.

The degradation of cell components is critical for the establishment of the physicochemical properties of the sebum and important for its interactions with bacteria. The breakdown of DNA decreases viscosity of liquids. Extracellular DNA released from skin cells is able to form complexes with antimicrobial peptides, leading to a decrease in their antibacterial and antiviral activity [145]. DNA is also a component of biofilms, and endogenous enzymes such as DNase1L2, which is highly expressed in the sebaceous gland [146], suppress biofilm formation [147]. Importantly, biofilm formation involving extracellular DNA is implicated in the pathogenesis by *Cutibacterium acnes* [148]. Yet, the contributions of host DNA and DNases to acne are not known at present.

The differentiation-associated breakdown of intracellular material may be a strategy to eradicate infectious material (viruses, endogenous retroviral elements, intracellular bacteria), but it may also be utilized by infectious agents for their spread [149]. Sebaceous glands have been identified as sites of bacterial and viral replication [150,151]. However, the role of holocrine secretion in the control of infections remains to be investigated.

DNase2-mediated degradation of nuclear DNA during terminal sebocyte differentiation leads to the generation and release of uric acid as a component of the sebum [56]. Since uric acid has been reported to function as a potent antioxidant and radical scavenger [152,153], programmed DNA degradation in sebocytes contributes to the maintenance of redox homeostasis at the skin surface. The breakdown of nucleic acids and proteins and the subsequent reactions provide a broad spectrum of metabolites, some of which are nutrients for the skin microbiome. Three genes implicated in the metabolism and transport of iron, namely *HMOX1*, *SLC40A1* and *HEPHL1* [154], are upregulated in differentiated sebocytes (SEB3 cell population) [72], suggesting that iron may be recycled prior to sebocyte programmed cell death. Autophagy, which has been discussed as a regulator of intracellular remodeling during sebocyte differentiation (Section 4.6), also affects the cellular metabolism and itself responds to both endogenous and exogenous stimuli [155,156,157]. The pathophysiological implications of holocrine secretion and the preceding metabolic processes are still emerging.

## 7. Conclusions and Perspectives

In conclusion, holocrine secretion is a mode of programmed cell death that is essential for the function of sebaceous glands. Mechanistically, holocrine death involves lysosomal enzymatic activity and rupture of the cell membrane. Although regulatory roles of autophagy and specific steps in DNA degradation during terminal differentiation of sebocytes have been characterized, many aspects of this important process remain to be determined.

The following unresolved questions should be addressed: 1. How do lysosomes contribute to sebocyte cell death? The activity labeling of lysosomal enzymes such as acid phosphatase [158] can be used to localize intact lysosomes in sebaceous glands and to infer their rupture by the loss of labeling. Immunolocalization of lysosomal proteins is an approach to test the hypothesis of their release into the cytoplasm. Targeted deletions of genes for lysosomal enzymes such as cathepsins in mouse models appears to be a promising approach for deciphering the molecular mechanisms of lysosome-dependent degradation in sebocytes. 2. How is the rupture of membranes controlled during holocrine secretion? The deletion of candidate pore proteins, such as GSDMA and ninjurin, in sebocytes of mice and in sebaceous gland models in vitro can be used to determine their roles. 3. Are desmosomes actively degraded to detach cell remnants during holocrine secretion? This question may be addressed by the deletion of candidate proteases or by applying protease inhibitors of different specificity in sebaceous gland models, followed by the study of cell fragments at sites of holocrine secretion. It will also be interesting to investigate potential aberrations of this process in diseases of sebaceous glands. Addressing these and other open questions will define the molecular control of programmed cell death in holocrine glands with potential implications for skin care and the dermatological management of highly prevalent skin diseases.

## Data Availability

No new data were created or analyzed in this study.
